# Examining the Role of Large Language Models in Orthopedics: Systematic Review

**DOI:** 10.2196/59607

**Published:** 2024-11-15

**Authors:** Cheng Zhang, Shanshan Liu, Xingyu Zhou, Siyu Zhou, Yinglun Tian, Shenglin Wang, Nanfang Xu, Weishi Li

**Affiliations:** 1 Department of Orthopaedics Peking University Third Hospital Beijing China; 2 Engineering Research Center of Bone and Joint Precision Medicine Ministry of Education Beijing China; 3 Beijing Key Laboratory of Spinal Disease Research Beijing China; 4 Peking University Health Science Center Beijing China

**Keywords:** large language model, LLM, orthopedics, generative pretrained transformer, GPT, ChatGPT, digital health, clinical practice, artificial intelligence, AI, generative AI, Bard

## Abstract

**Background:**

Large language models (LLMs) can understand natural language and generate corresponding text, images, and even videos based on prompts, which holds great potential in medical scenarios. Orthopedics is a significant branch of medicine, and orthopedic diseases contribute to a significant socioeconomic burden, which could be alleviated by the application of LLMs. Several pioneers in orthopedics have conducted research on LLMs across various subspecialties to explore their performance in addressing different issues. However, there are currently few reviews and summaries of these studies, and a systematic summary of existing research is absent.

**Objective:**

The objective of this review was to comprehensively summarize research findings on the application of LLMs in the field of orthopedics and explore the potential opportunities and challenges.

**Methods:**

PubMed, Embase, and Cochrane Library databases were searched from January 1, 2014, to February 22, 2024, with the language limited to English. The terms, which included variants of “large language model,” “generative artificial intelligence,” “ChatGPT,” and “orthopaedics,” were divided into 2 categories: *large language model* and *orthopedics*. After completing the search, the study selection process was conducted according to the inclusion and exclusion criteria. The quality of the included studies was assessed using the revised Cochrane risk-of-bias tool for randomized trials and CONSORT-AI (Consolidated Standards of Reporting Trials–Artificial Intelligence) guidance. Data extraction and synthesis were conducted after the quality assessment.

**Results:**

A total of 68 studies were selected. The application of LLMs in orthopedics involved the fields of clinical practice, education, research, and management. Of these 68 studies, 47 (69%) focused on clinical practice, 12 (18%) addressed orthopedic education, 8 (12%) were related to scientific research, and 1 (1%) pertained to the field of management. Of the 68 studies, only 8 (12%) recruited patients, and only 1 (1%) was a high-quality randomized controlled trial. ChatGPT was the most commonly mentioned LLM tool. There was considerable heterogeneity in the definition, measurement, and evaluation of the LLMs’ performance across the different studies. For diagnostic tasks alone, the accuracy ranged from 55% to 93%. When performing disease classification tasks, ChatGPT with GPT-4’s accuracy ranged from 2% to 100%. With regard to answering questions in orthopedic examinations, the scores ranged from 45% to 73.6% due to differences in models and test selections.

**Conclusions:**

LLMs cannot replace orthopedic professionals in the short term. However, using LLMs as copilots could be a potential approach to effectively enhance work efficiency at present. More high-quality clinical trials are needed in the future, aiming to identify optimal applications of LLMs and advance orthopedics toward higher efficiency and precision.

## Introduction

### Background

Large language models (LLMs) typically refer to pretrained language models (PLMs) that have a large number of parameters and are trained on massive amounts of data. In recent years, this area has emerged as one of the most prominent areas of research in artificial intelligence (AI) innovation [1,2]. What makes LLMs different from smaller-scale PLMs is their remarkable emergent abilities to solve complex tasks. Studies have found that LLMs, such as generative pretrained transformer (GPT)-3 with approximately 175 billion parameters, exhibit a significant leap in natural language processing (NLP) capabilities compared to PLMs with fewer parameters, such as GPT-2 with approximately 1.5 billion parameters [2,3]. Generative AI applications developed based on LLMs not only possess the ability to understand natural language but can also generate corresponding text, images, and even videos based on input sources. This human-machine interaction mode holds great potential in medical scenarios.

LLMs have undergone significant advancements in recent years; currently, the most prevalent web-based LLM service is ChatGPT (OpenAI). Launched in November 2022, ChatGPT is a chatbot application developed based on GPT-3.5 or GPT-4 after fine-tuning, and it can quickly respond to questions posed by users. In addition to ChatGPT, applications include Bard (upgraded to Gemini in December 2023) based on Language Model for Dialogue Applications (Google LLC); Med-PaLM 2 (Google LLC); ERNIE Bot (Baidu); and MOSS (Fudan University). GPT-4 can approach or achieve human-level performance in cognitive tasks across various fields, including medical domains [4]. When answering the 2022 United States Medical Licensing Examination questions, without further training or reinforcement, ChatGPT reached or approached a passing level in all 3 examinations [5]. However, answering examination questions does not directly reflect the performance of LLMs in clinical applications. The value and safety of a chatbot that is already in use are still not fully understood, making clinical research both essential and imperative. Published narrative reviews and editorials have explored the medical applications of LLM technology from 3 perspectives: clinical practice, education, and research [1,6-8]. These publications also provide a preliminary assessment of the value and safety of LLMs, offering guidance for exploring their use in specialized medical fields.

Orthopedics is a significant branch of medicine, typically encompassing disciplines such as trauma, spine surgery, joint surgery, sports medicine, hand surgery, and bone oncology. Orthopedic diseases have a broad impact on populations and pose a major global health threat. Low back pain, a common symptom in orthopedics or spine surgery, has been identified as the leading cause of global productivity loss, as measured in years, according to a large-scale epidemiological study covering 195 countries and regions; in 126 countries, low back pain ranks first among the causes of years lived with disability [9]. In traditional health care systems, the annual medical expenditure for low back pain in the United States is estimated to exceed US $100 billion, contributing to a significant socioeconomic burden [10]. Similarly, osteoarthritis is also a critical global health issue. The global prevalence of knee osteoarthritis in adults aged >40 years is 23%, with approximately 61% of adults aged >45 years showing radiographic evidence of knee osteoarthritis [11]. Therefore, applying LLMs in orthopedics holds the potential to alleviate the current heavy socioeconomic burden.

It is worth noting that several pioneers in orthopedics have conducted studies on LLMs across various subspecialties to explore their performance in addressing different issues. However, there are currently few reviews and summaries of these studies. The published reviews primarily focus on introducing and popularizing the basic concepts of LLMs in orthopedics [12,13], or they offer forward-looking perspectives by categorizing LLM applications in clinical practice, education, and research [14]. A systematic summary of existing research is absent. To the best of our knowledge, this review is the first to systematically summarize existing research findings. In contrast to prior works, we place greater emphasis on the quantitative evaluation methods and results of these studies because we believe that these methods and outcomes can help orthopedic and computer science researchers better understand the current state of LLM research and the performance of LLMs. Regarding application categorization, we consider tasks involving NLP in management as another important application area for LLMs in orthopedics. Therefore, this review adds a category for orthopedic management applications to the existing classification framework.

### Objectives

The objective of this review was to comprehensively summarize the research findings on the application of LLMs in orthopedics and outline the advantages, limitations, and methodological evaluations, while also exploring the potential opportunities and challenges emerging in this era, for facilitating interdisciplinary collaboration and advancement among researchers in computer science and orthopedics. The ultimate goal is to contribute to improved efficiency and quality of orthopedic care as well as a reduction in medical costs and the associated socioeconomic burden.

## Methods

### Search Strategy

The protocol for this systematic review followed the PRISMA (Preferred Reporting Items for Systematic Reviews and Meta-Analyses) guidelines (checklist can be found in the Multimedia Appendix 1) [15]. PubMed, Embase, and Cochrane Library databases were searched, with the language limited to English. The time frame was set from January 1, 2014, to February 22, 2024. Search terms were divided into 2 categories, with the first category including LLM-related terms and the second containing words related to orthopedics and its subspecialties (Textbox 1). Terms within each category were connected using “OR,” while terms within different categories were connected using “AND.” The full search strategy can be found in Multimedia Appendix 2.

**Categories and terms applied in the search queries.**

**Category 1**

- “large language model,” “LLM,” “generative artificial intelligence,” “generative AI,” “ChatGPT,” and “Generative Pre-Trained Transformer”

**Category 2**

- “orthopedics,” “bone,” “musculoskeletal,” “injury,” “wound,” “trauma,” “articular,” “joint,” “sports medicine,” “hand surgery,” “spine,” “spinal, “cervical vertebrae,” “thoracic vertebrae,” “lumbar vertebrae,” “sacrum,” “coccyx,” “spinal canal,” “vertebral body,” and “intervertebral disc”

### Study Selection

The records were downloaded from the databases and imported into EndNote (version 21.2; Clarivate) for article management. The study selection process was conducted independently by 2 investigators (CZ and SL). The inclusion and exclusion criteria are listed in Textbox 2. The results were cross-checked, and discrepancies were resolved through discussion, with the final determination made by a third investigator (YT).

**Inclusion and exclusion criteria.**

**Inclusion criteria**

- Article type
  Original research
- Language
  Articles written in English
- Content
  Studies that use at least 1 large language model (LLM)
  Studies that are relevant to the field of orthopedics

**Exclusion criteria**

- Article type
  Reviews, editorials, letters, and study protocols
- Language
  Articles written in a language other than English
- Content
  Studies that do not involve LLMs
  Studies that use LLMs for tasks such as code generation, debugging, or text generation without any performance evaluation of the model

### Quality Assessment of Studies

Quality assessment was conducted by 2 investigators (CZ and SL) independently. First, the study designs were identified. Studies that involved only posing questions to LLMs, did not recruit participants, and did not report a study design were classified as surveys. Given the diverse nature of the survey types included in the review, quality assessments were conducted only for studies that recruited participants. The revised Cochrane risk-of-bias tool for randomized trials [16] was used to assess randomized controlled trials (RCTs), and the CONSORT-AI (Consolidated Standards of Reporting Trials–Artificial Intelligence) guidance [17] was used to evaluate prospective or retrospective observational studies. The revised Cochrane risk-of-bias tool (version of August 22, 2019) is designed for assessing RCTs and contains 5 domains: bias arising from the randomization process, bias due to deviations from the intended interventions, bias due to missing outcome data, bias in measurement of the outcome, and bias in selection of the reported result. The CONSORT-AI guidance is a new reporting guideline specifically designed for clinical trials that assess interventions with an AI component. The quality assessment domains under this guidance include a statement of the AI algorithm used, details of how the AI intervention fits within the clinical pathway, inclusion and exclusion criteria for input data, a description of the approaches used to handle unavailable input data, a description of the input data acquisition process for the AI intervention, specifications of human-AI interaction in the collection of input data, the output of the AI algorithm, and explanations of how the AI intervention’s outputs contribute to health behavior changes. The results were cross-checked, and discrepancies were resolved through discussion, with the final determination made by another investigator (YT).

### Data Extraction and Synthesis

The studies were categorized into 4 groups based on their application areas: clinical practice, education, research, and management. Data extraction and synthesis were conducted by 2 investigators (CZ and SL) independently. In addition to general characteristics, the composition of extracted data varied depending on the specific category. Details of the data extraction strategy can be found in Multimedia Appendix 3. In cases where there were inconsistencies in the process, a third investigator (XZ) participated in the discussion and made the final decision. For studies with high heterogeneity, we did not synthesize the parameters for model performance evaluation and instead focused on providing a descriptive analysis of the data. Microsoft Excel 2019 was used for data collection, analysis, and visualization.

## Results

### Characteristics of Included Studies

**Figure 1:**
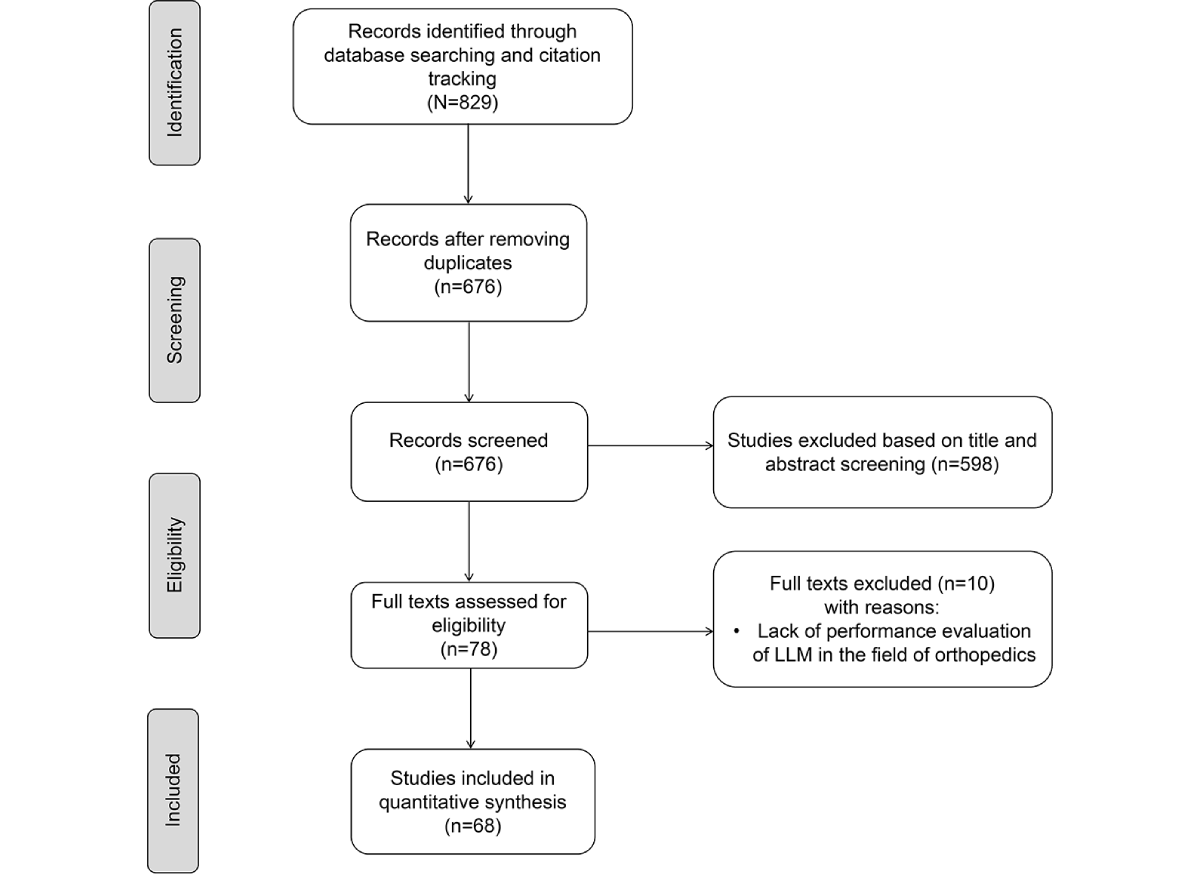
Flowchart of literature screening based on the PRISMA (Preferred Reporting Items for Systematic Reviews and Meta-Analyses) statement. LLM: large language model.

A total of 829 studies were identified; after removing duplicates and screening, 68 (8.2%) studies were selected in the literature review. The inclusion process is shown in Figure 1.

**Figure 2:**
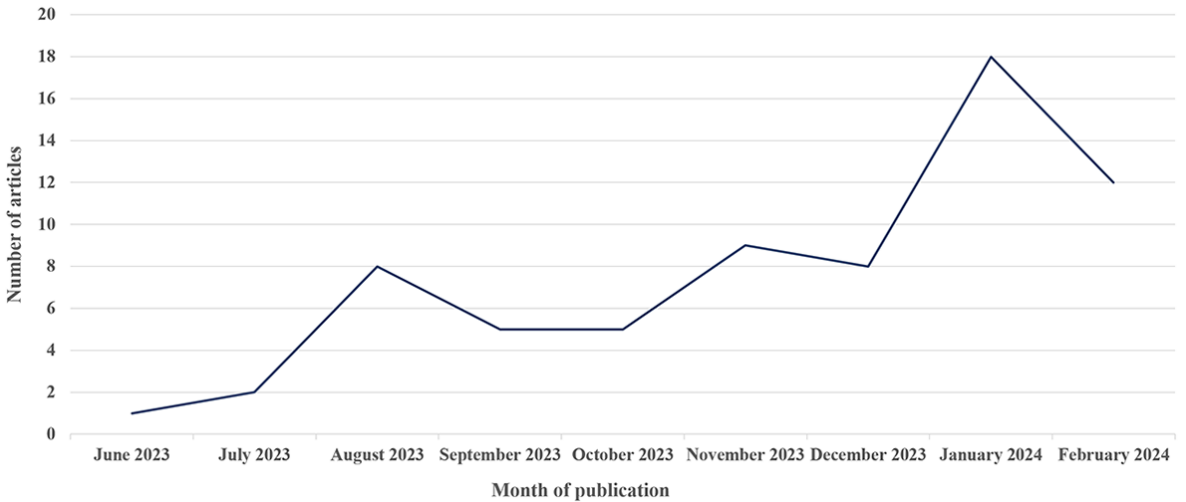
Trends in the number of publications.

The application of LLMs in orthopedics involves the fields of clinical practice, education, research, and management. Of the 68 included studies, 47 (69%) focused on clinical practice (Table 1) [18-64], 12 (18%) addressed orthopedic education (Table 2) [65-76], 8 (12%) were related to scientific research (Table 3) [77-84], and 1 (1%) pertained to the field of management (Table 3) [85]. Of the 68 studies, 55 (81%) were classified as surveys; furthermore, only 8 (12%) recruited patients, only 1 (1%) was a high-quality study (RCT), and only 1 (1%) was a prospective study. Since June 2023, research on the application of LLMs in orthopedics has increased month by month (Figure 2).

**Table 1:** Characteristics of the included studies focused on clinical practice.

Study, year	Study design	Task	LLM^a^ tools	Main evaluation metrics for model performance and their values	Enrolled participants, n	Subjective or objective assessment of the model’s performance
Agharia et al [18], 2024	Survey	Formulate clinical decisions	GPT-3.5; GPT-4; Bard	Proportion of most popular response: 68% (GPT-4); 40.2% (GPT-3.5); 45.4% (Bard)	—^b^	Subjective
Anastasio et al [19], 2023	Survey	Generate answers to clinical questions	GPT-3.5	Ratio of responses in different quality grades: bottom-tier rating 4.5%; middle-tier rating 27.3%; top-tier rating 68.2%	—	Subjective
Baker et al [20], 2024	RCT^c^	Assist with writing patient histories	GPT-4	Mean time: 69.8 (SD 26.2) s; mean word count: 135.8 (SD 40.3); mean PDQI-9^d^ score: 35.6 (SD 3.1); mean overall rating: 3.8 (SD 0.6); ratio of erroneous documents: 36%	11	Subjective
Christy et al [21], 2024	Survey	Generate answers to clinical questions	GPT-3.5	Ratio of appropriate responses in total responses: 78%; intraclass correlation coefficient: 0.12	—	Subjective
Coraci et al [22], 2023	Cross-sectional study	Create questionnaire for assessment	GPT-3.5	Correlation: acceptable correlation with ODI^e^ and QBPDS^f^; no statistical correlation with RMDQ^g^ or NRS^h^	20	Subjective
Crook et al [23], 2023	Survey	Generate answers to clinical questions	GPT-3	DISCERN score: 58; JAMA^i^ benchmark score: 0/4; FRE^j^ score: 34; FKGL^k^ score: 15	—	Subjective
Daher et al [24], 2023	Prospective study	Diagnose and manage patients	GPT-3	Accuracy of diagnosis: 93%; accuracy of management: 83%	29	Objective
Decker et al [25], 2023	Cross-sectional study	Generate informed consent documentation	GPT-3.5	Mean readability, accuracy, and completeness scores (surgeons vs LLMs): readability= 15.7 vs 12.9; risks=1.7 vs 1.7; benefits=1.4 vs 2.3; alternatives=1.4 vs 2.7; overall impression=1.9 vs 2.3; composite: 1.6 vs 2.2	—	Subjective
Draschl et al [26], 2023	Survey	Generate answers to clinical questions	GPT-3.5	5-point Likert scores, mean: completeness=3.80 (SD 0.63); misleading=4.04 (SD 0.67); errors=4.14 (SD 0.58); up-to-dateness=3.90 (SD 0.45); suitability for patients=3.69 (SD 0.64); suitability for surgeons=3.63 (SD 0.95)	—	Subjective
Dubin et al [27], 2023	Survey	Generate answers to clinical questions	GPT-3	25% of the questions were similar when performing a Google web search and a search of ChatGPT for all search terms; 75% of the questions were answered by government websites; 55% of the answers were different between Google web search and ChatGPT in terms of numerical questions	—	Subjective
Duey et al [28], 2023	Survey	Generate answers to clinical questions	GPT-3.5; GPT-4	Accuracy: 33% (GPT-3.5); 92% (GPT-4)	—	Subjective
Fabijan et al [29], 2023	Cross-sectional study	Classify cases of single-curve scoliosis	GPT-4; Microsoft Bing with GPT^l^; Scholar AI Premium	GPT-4 and Scholar AI Premium excelled in classifying single-curve scoliosis with perfect sensitivity (100%) and specificity (100%)	56	Objective
Fahy et al [30], 2024	Survey	Generate answers to clinical questions	GPT-3.5; GPT-4	GPT-3.5 vs GPT-4 mean DISCERN score: 55.4 vs 62.09; mean reading grade level score: 18.08 vs 17.90	—	Subjective
Gianola et al [31], 2024	Survey	Generate answers to clinical questions	GPT-3.5	Internal consistency: 49%; accuracy: 33%	—	Subjective
Hurley et al [32], 2024	Survey	Generate answers to clinical questions	ChatGPT	DISCERN score: 60; JAMA benchmark score: 0; FRE score: 26.2; FKGL score: considered to be that of a college graduate	—	Subjective
Johns et al [33], 2024	Survey	Generate answers to clinical questions	GPT-3.5	Satisfaction rate: 60%	—	Subjective
Johns et al [34], 2024	Survey	Generate answers to clinical questions	GPT-3.5	DISCERN score: 41; FKGL score: 13.4; satisfaction rate: 40%	—	Subjective
Kaarre et al [35], 2023	Survey	Generate answers to clinical questions	GPT-4	Average correctness of responses for patients and physicians: 1.69 and 1.66, respectively (on a scale ranging from 0=incorrect, 1=partially correct, and 2=correct)	—	Subjective
Kasthuri et al [36], 2024	Survey	Generate answers to clinical questions	Microsoft Bing with GPT-4	Mean completeness score: 2.03; mean accuracy score: 4.49	—	Subjective
Kienzle et al [37], 2024	Survey	Generate answers to clinical questions	GPT-4	Mean DISCERN score in overall quality: 3.675	—	Subjective
Kirchner et al [38], 2023	Survey	Rewrite patient education materials	GPT-3.5	Mean FKGL score in patient education materials related to herniated lumbar disk, scoliosis, stenosis, TKA^m^, and THA^n^: before rewrite=9.5, 12.6, 10.9, 12.0, and 6.3, respectively; after rewrite=5.0, 5.6, 6.9, 11.6, and 6.1, respectively	—	Subjective
Kuroiwa et al [39], 2023	Survey	Generate answers to clinical questions	GPT-3.5	Ratios of correct answers: 25/25, 1/25, 24/25, 16/25, and 17/25 for carpal tunnel syndrome, cervical myelopathy, lumbar spinal stenosis, knee osteoarthritis, and hip osteoarthritis, respectively	—	Objective
Li et al [40], 2023	Survey	Generate answers to clinical questions	GPT-4	Mean accuracy score (out of 5): 4.3; mean completeness score (out of 3): 2.8	—	Subjective
Li et al [41], 2024	Survey	Generate answers to clinical questions	GPT-3.5	1 response was excellent, requiring no clarification; 4 responses were satisfactory, requiring minimal clarification; 3 responses were satisfactory, requiring moderate clarification; 2 responses were unsatisfactory	—	Subjective
Lower et al [42], 2023	Survey	Deliver safe and coherent medical advice	GPT-4	Mean Likert scale score: 3.2	—	Subjective
Magruder et al [43], 2024	Survey	Generate answers to clinical questions	ChatGPT	Answer grades (from 1 to 5), mean: relevance=4.43 (SD 0.77); clarity=4.22 (SD 0.86); accuracy=4.10 (SD 0.90); evidence based=3.92 (SD 1.01); completeness=3.91 (SD 0.88); consistency=3.54 (SD 1.10)	—	Subjective
Mika et al [44], 2023	Survey	Generate answers to clinical questions	GPT-3.5	2 responses were excellent, requiring no clarification; 4 responses were satisfactory, requiring minimal clarification; 3 responses were satisfactory, requiring moderate clarification; 1 response was unsatisfactory	—	Subjective
Pagano et al [45], 2023	Retrospective observational study	Formulate diagnosis and potential treatment suggestions	GPT-4	Diagnostic accuracy: 100% for the total cases; concordance in therapeutic recommendations: 83% for the total cases	100	Objective
Mejia et al [46], 2024	Survey	Generate answers to clinical questions	GPT-3.5; GPT-4	Accuracy: 52% (GPT-3.5); 59% (GPT-4); overconclusiveness: 48% (GPT-3.5); 45% (GPT-4)	—	Subjective
Russe et al [47], 2023	Retrospective observational study	Provide accurate fracture classification based on radiology reports	FraCChat; GPT-3.5-Turbo; GPT-4	Accuracy: GPT 3.5=3%; GPT 4=2%; FraCChat 3.5=48%; FraCChat 4=71%	—	Objective
Schonfeld et al [48], 2024	Retrospective cohort study	Predict outcome of adult spinal deformities	Gatortron	AUC^o^ scores: 0.565 (pulmonary complication); 0.559 (neurological complication); 0.557 (sepsis); 0.508 (delirium); *F*_1_-scores: 0.545 (pulmonary complication); 0.250 (neurological complication); 0.383 (sepsis); 0.156 (delirium)	209	Objective
Seth et al [49], 2023	Survey	Generate answers to clinical questions	ChatGPT	Mean Likert scale score: 3.1	—	Subjective
Shrestha et al [50], 2024	Survey	Generate answers to clinical questions	GPT-3.5	Accuracy: 44%-65% for different guideline variations	—	Subjective
Sosa et al [51], 2024	Survey	Generate answers to clinical questions	GPT-4; Bard; Bing AI	Ratios of appropriate answers to questions related to bone physiology: 83.3% (GPT-4); 23.3% (Bing AI); 16.7% (Bard)	—	Subjective
Stroop et al [52], 2023	Survey	Generate answers to clinical questions	ChatGPT	Ratio of medically complete correct answers: 52%; ratio of medically complete and comprehensive answers: 55%	—	Subjective
Suthar et al [53], 2023	Retrospective observational study	Generate diagnosis	GPT-4	Accuracy rate in spine cases: 55%	—	Objective
Taylor et al [54], 2024	Survey	Generate answers to clinical questions	ChatGPT	Ratio of surgeons who reported that the questions had been appropriately answered: 91%	—	Subjective
Temel et al [55], 2024	Survey	Generate answers to clinical questions	GPT-4	Ensuring Quality Information for Patients score: mean 43.02 (SD 6.37); FRE score: mean 26.24 (SD 13.81); FKGL score: mean 14.84 (SD 1.79)	—	Subjective
Tharakan et al [56], 2024	Survey	Generate answers to clinical questions	GPT-3	Answers provided by ChatGPT cited more academic references than those provided by a Google search (80% vs 50%)	—	Subjective
Truhn et al [57], 2023	Retrospective observational study	Prioritize treatment recommendations	GPT-4	The overall quality of the treatment recommendations was rated as good or better	20	Subjective
Warren et al [58], 2024	Survey	Generate answers to clinical questions	GPT-3.5	Answers to fact, policy, and value questions (mean scores): DISCERN=51, 53, and 55, respectively; JAMA benchmark=0, 0, and 0, respectively; FRE=48.3, 42.0, and 38.4, respectively; FKGL=10.3, 10.9, and 11.6, respectively	—	Subjective
Wilhelm et al [59], 2023	Survey	Generate treatment recommendations	Claude-instant-v1.0; GPT 3.5-Turbo; Command-xlarge-nightly; Bloomz	Mean DISCERN quality scores: 3.4 (Claude-instant-v1.0); 2.8 (GPT 3.5-Turbo); 2.2 (Command-xlarge-nightly); 1.1 (Bloomz)	—	Subjective
Wright et al [60], 2024	Survey	Generate answers to clinical questions	GPT-3.5	Mean accuracy score: 4.26; mean comprehensiveness score: 3.79	—	Subjective
Yang et al [61], 2024	Retrospective observational study	Generate diagnosis	GPT-3.5	Accuracy: 0.87; sensitivity: 0.99; specificity: 0.73	1366	Objective
Yang et al [62], 2024	Survey	Generate answers to clinical questions	ChatGPT; Bard	Concordance with the AAOS^p^ Clinical Practice Guidelines: 80% (ChatGPT); 60% (Bard)	—	Subjective
Yapar et al [63], 2024	Survey	Generate answers to clinical questions	GPT-4	Accuracy: 79.8%; applicability: 75.2%; comprehensiveness: 70.6%; communication clarity: 75.6%	—	Subjective
Zhou et al [64], 2024	Case study	Generate answers to clinical questions related to the case	GPT-3.5	No statistical results	—	Subjective

^a^LLM: large language model.

^b^Not applicable.

^c^RCT: randomized controlled trial.

^d^PDQI-9: Physician Documentation Quality Instrument-9.

^e^ODI: Oswestry Disability Index.

^f^QBPDS: Quebec Back Pain Disability Scale.

^g^RMDQ: Roland-Morris Disability Questionnaire.

^h^NRS: numerical rating scale.

^i^JAMA: Journal of the American Medical Association.

^j^FRE: Flesch reading ease.

^k^FKGL: Flesch-Kincaid grade level.

^l^GPT: generative pretrained transformer.

^m^TKA: total knee arthroplasty.

^n^THA: total hip arthroplasty.

^o^AUC: area under the curve.

^p^AAOS: American Academy of Orthopaedic Surgeons.

**Table 2:** Characteristics of the included studies focused on orthopedic education.

Study, year	Study design	Task	LLM^a^ tools	Source	Questions, n	Scores or accuracy (%)
Cuthbert and Simpson [65], 2023	Survey	Examination	GPT-3.5	UKITE^b^	134	35.8
Ghanem et al [66], 2023	Survey	Examination	GPT-4	OITE^c^	201	61.2
Han et al [67], 2024	Survey	Examination	GPT-3.5	ASSH^d^	1583	36.2
Hofmann et al [68], 2023	Survey	Examination	GPT-3.5; GPT-4	OITE	410 (GPT-3.5); 396 (GPT-4)	GPT-3.5: 46.3; GPT-4: 63.4
Jain et al [69], 2024	Survey	Examination	GPT-3.5	OITE	360	52.8
Kung et al [70], 2023	Survey	Examination	GPT-3.5; GPT-4	OITE	360	GPT-3.5: 54.3; GPT-4: 73.6
Lum [71], 2023	Survey	Examination	GPT-3.5	OITE	207	47
Massey et al [72], 2023	Survey	Examination	GPT-3.5; GPT-4	ResStudy Orthopaedic Examination Question Bank	180	GPT-3.5: 29.4; GPT-4: 47.2
Ozdag et al [73], 2023	Survey	Examination	GPT-3.5	OITE	102	45
Rizzo et al [74], 2024	Survey	Examination	GPT-3.5-Turbo; GPT-4	OITE	2022: 207; 2021: 213; 2020: 215	2022: GPT-4=67.63; GPT 3.5-Turbo=50.24; 2021: GPT-4=58.69; GPT 3.5-Turbo=47.42; 2020: GPT-4=59.53; GPT 3.5-Turbo=46.51
Saad et al [75], 2023	Survey	Examination	GPT-4	Mock FRCS Orth^e^ Part A	240	67.5
Traoré et al [76], 2023	Survey	Examination	GPT-3.5	EBHS^f^ diploma examination	18	0

^a^LLM: large language model.

^b^UKITE: United Kingdom and Ireland In-Training Examination.

^c^OITE: Orthopaedic Surgery In-Training Examination.

^d^ASSH: American Society for Surgery of the Hand.

^e^FRCS Orth: Orthopaedic Fellow of the Royal College of Surgeons.

^f^EBHS: European Board of Hand Surgery.

**Table 3:** Characteristics of the included studies focused on orthopedic research and management.

Study, year	Study design	Task	LLM^a^ tools	Input	Key findings
Gill et al [77], 2024	Survey	Improve readability	GPT-3.5	IRB^b^-approved orthopedic surgery research consent forms	ChatGPT can significantly improve the readability of orthopedic clinical research consent forms; 63.2% of the post-ChatGPT consent forms had at least 1 error
Hakam et al [78], 2024	Survey	AI^c^-Generated scientific literature	GPT-3.4; You.com	Five abstracts about meniscal injuries	The AI-generated texts could not be successfully identified
Kacena et al [79], 2024	Survey	Write scientific review articles	GPT-4	Prompts	AI reduced the time for writing but had significant inaccuracies
Lawrence et al [80], 2024	Survey	Generate abstract	GPT-3	A standard set of input commands	Interrater reliability for abstract quality scores was moderate
Lotz et al [81], 2023	Survey	Assist new research hypothesis exploration	Toolkit based on GPT-3.5	Prior studies	LLMs may be useful for analyzing and distinguishing publications, as well as determining the degree to which the literature supports or contradicts emergent hypotheses
Methnani et al [82], 2023	Survey	Calculate sample size	GPT-3.5	All necessary data, such as mean, percentage SD, normal deviations, and study design	In 1 (25%) of the 4 trials, the sample size was correctly calculated
Nazzal et al [83], 2024	Survey	Write a review article	GPT-4	Prompts	The AI-only paper was the most inaccurate, with inappropriate reference use, and the AI-assisted paper had the greatest incidence of plagiarism
Sanii et al [84], 2023	Survey	Perform an orthopedic surgery literature review	GPT-3; Perplexity	Standard prompts	The current iteration of ChatGPT cannot perform a reliable literature review, and Perplexity is only able to perform a limited review of the medical literature
Zaidat et al [85], 2023	Retrospective cohort study	Predict CPT^d^ codes	GPT-4	Surgical operative notes	The AUROC^e^ score was 0.87, and the AUPRC^f^ score was 0.67

^a^LLM: large language model.

^b^IRB: institutional review board.

^c^AI: artificial intelligence.

^d^CPT: current procedural terminology.

^e^AUROC: area under the receiver operating characteristic curve.

^f^AUPRC: area under the precision-recall curve.

### Quality Assessment of Studies

We conducted quality assessments for the 8 studies that recruited participants (Multimedia Appendices 4 and 5). The RCT study was evaluated using the revised Cochrane risk-of-bias tool for randomized trials, and it was found to have a low risk of bias in all 5 domains—bias arising from the randomization process, bias due to deviations from the intended interventions, bias due to missing outcome data, bias in measurement of the outcome, and bias in selection of the reported result—indicating high study quality. The remaining studies (7/8, 88%; observational studies) were evaluated using the CONSORT-AI guidance and were found to be of good quality.

### Distribution of LLM Tools

Among all the LLM tools applied, ChatGPT was the most commonly mentioned. Other LLM tools included Bard, Microsoft Bing, Scholar AI, Perplexity, Gatortron, Claude, Command-xlarge-nightly, and Bloomz, as well as software developed by researchers based on commonly used LLM kernels. Currently, there are 2 main versions of ChatGPT available: GPT-3 (including GPT-3.5 and GPT-3.5-Turbo) and GPT-4. Most of the studies (48/68, 71%) specified the version of the tool used. The majority of the studies (25/48, 52%) only used GPT-3 or 3.5, likely due to the publication lag because these studies were conducted before the release of GPT-4. Given that GPT-4 outperforms GPT-3 in most tasks, future research should primarily use GPT-4.

### Model Performance Evaluation

As shown in Tables 1-3, there is considerable heterogeneity in the definition, measurement, and evaluation of LLM performance across the included studies. Currently, there is no unified research paradigm for the application of LLMs in medicine. Therefore, this review focused on different model performance evaluation metrics according to various application categories. For clinical applications of LLMs, we were particularly concerned with the accuracy of model reasoning and the readability of the generated text; unfortunately, the majority of the studies (39/47, 83%) relied on subjective assessments of the model’s performance. In studies with objective evaluations, the heterogeneity in the subtasks performed by the LLMs (including diagnosis, classification, clinical case analysis, and case text generation) prevented us from pooling the data. For diagnostic tasks alone, the accuracy ranged from 55% to 93% [24,53]. When performing disease classification tasks, GPT-4’s accuracy ranged from 2% to 100% [29,47] (Table 1). In studies on readability, the most commonly used metrics are the Flesch Reading Ease and the Flesch-Kincaid Grade Level (FKGL) scores. The FKGL metric correlates reading difficulty with years of education, providing a straightforward reflection of the readability of generated materials. In these studies, the generated texts had FKGL scores ranging from a minimum of 5.0 [38], indicating primary school reading difficulty, to the maximum required years of education [32], showing significant variability. This variability is likely due to differences in the research questions, methodologies, prompts, and evaluators. In the educational applications (eg, answering questions from examination papers), the most frequently used test source (7/12, 58%) was the Orthopaedic Surgery In-Training Examination (OITE). The test scores are widely recognized as performance evaluation metrics for the models, with final scores ranging from 45% to 73.6% due to differences in models and test selections (Table 2). For applications of LLMs in research and management, the flexible and varied nature of the tasks led to substantial differences in performance measurement and evaluation. Therefore, we collected the model inputs and major findings for a descriptive presentation (Table 3).

## Discussion

### Overview

Despite the relatively short time since their introduction and the absence of rigorous and comprehensive performance evaluation in highly specialized fields such as orthopedics, it is an undeniable fact that LLMs have already been made accessible to the public. Given the increasing acceptance and widespread adoption of LLMs, it is imperative for orthopedic surgeons to possess a comprehensive understanding of their operational mechanisms and limitations. Users should also delineate the safe application boundaries while harnessing the benefits offered by LLMs, all while mitigating potential risks in their daily clinical practice. This section presents a comprehensive overview of application examples and model performance across diverse fields, while providing strategic approaches to address LLMs based on our findings. In addition, in this section, we critically evaluate research methodologies and offer potential recommendations for future investigations.

### Application of LLMs in the Field of Orthopedic Education

LLMs can not only provide answers but also offer explanations and even engage in further discussions on a given topic, demonstrating potential value in orthopedic education. Several studies have evaluated the performance of ChatGPT in answering questions related to orthopedics and further discussed its value in the field of orthopedic education. The source of questions includes the OITE [66,68-71,73,74], the ResStudy Orthopaedic Examination Question Bank [72], the Fellowship of the Royal College of Surgeons (Trauma and Orthopaedic Surgery) examination [65,75], and hand surgery examinations in the United States and Europe [67,76]. Accuracy (scores) and whether the answers meet the standard are important evaluation criteria for LLM performance. Another educational indicator is the correctness and reasonableness of answer explanations. Studies evaluating OITE questions usually convert accuracy into postgraduate year (PGY) levels for evaluation. Due to differences in software applications and question selection, different studies have reported varying performances of ChatGPT. ChatGPT with GPT-4 performed at an average level ranging from PGY-2 to PGY-5 [66,68,70,74], while ChatGPT with GPT-3.5 performed slightly better than PGY-1 or below the average level of PGY-1 [68-71,73,74]. For correct answers, ChatGPT can provide explanations and reasoning processes consistent with those of examiners, which helps students understand the questions and general orthopedic principles [66,69]. However, ChatGPT failed to pass the Fellowship of the Royal College of Surgeons (Trauma and Orthopaedic Surgery) examination and hand surgery examinations in the United States and Europe [65,67,75,76]. In addition, as a language model, ChatGPT cannot analyze medical images correctly [70], limiting its role in orthopedic imaging education.

Although LLMs currently cannot fully replace orthopedic instructors, they can still serve as a valuable supplementary tool for learning. Integrating their responses with authoritative resources for verification and using appropriate prompts can optimize their capacity to offer logical explanations and foster critical thinking.

### Application of LLMs in Clinical Practice

#### Medical Consultation and Physician-Patient Communication

One challenge faced by orthopedic physicians is that, unlike in the case of other clinical interventions, LLMs have already been integrated as medical consultation tools in the diagnosis and treatment process of numerous diseases without sufficient clinical evidence and regulatory review from authorities such as the US Food and Drug Administration. LLMs can be considered an alternative approach for patients who have sustained injuries or experience discomfort before seeking guidance from primary care physicians or specialists. When confronted with medical issues, individuals who rely heavily on the internet for problem-solving in their personal and professional lives often exhibit a tendency to seek treatment decisions on the web [30]. Compared to traditional search engines or Wikipedia, LLMs could potentially become a significant source of medical consultation information, especially in cases of nonacute diseases such as lower back pain or joint pain. Meanwhile, many physicians also hope that LLMs can help alleviate their burden of simple medical consultations and repetitive paperwork related to physician-patient communication (such as preoperative consent forms), which is considered 1 of the important factors contributing to physician burnout [86]. Although LLMs can provide concise, clarified, or simplified responses related to the given topic and deliver high-quality and empathetic answers [66,87], given their imperfect performance in addressing questions related to orthopedics [65-76], caution should be exercised regarding their reliability in orthopedic consultation scenarios.

Studies have evaluated the performance of LLMs in answering questions related to hand surgery [23], spinal cord injuries [55], joint and sports medicine [19,27,35,41,56,58], and preoperative physician-patient communication for lumbar disk herniation [52] and hip replacement surgery [44]. In these studies, the evaluation criteria of interest typically encompass the model’s answer accuracy, readability, completeness, and information sources. Evaluation methods often encompass scale assessments or subjective ratings conducted by researchers. The DISCERN score is commonly used to evaluate answer quality [19,23,58,88], while FKGL and Flesch Reading Ease scores are commonly used to measure readability [23,55,58]. The accuracy of LLMs’ responses is closely correlated with the specific topic. Questions in the field of joint and sports medicine often receive high-quality responses, while there are serious issues with the quality of answers regarding spinal cord injuries. There are also significant differences in the evaluation of the readability or comprehensibility of LLMs’ answers, with some researchers considering them to be easily understood [44,52], while studies using Flesch-related scales suggest that LLMs’ answers require a reading level of at least 10 years of education or even university level for full comprehension [23,55,58]. The underlying factors contributing to this phenomenon can be attributed to variations in question topics, prompts, and evaluation methodologies used for answer assessment. Consequently, orthopedic surgeons should exercise caution when interpreting the findings of these studies.

Although LLMs can offer more scholarly health information in comparison to search engines [27,56], they still cannot replace orthopedic physicians in medical consultation and physician-patient communication. Using LLMs as a guiding tool and maintaining communication with physicians during further diagnosis and treatment decisions may be a safer and more effective strategy.

#### Clinical Workflow

The performance of LLMs in orthopedic examinations suggests that they cannot handle complex tasks independently, but they hold potential to serve as valuable assistants for orthopedic physicians. One possible application is using LLMs to automate simple, repetitive tasks such as writing medical records for common orthopedic diseases [20]. In the context of complex disease management tasks, LLMs can possess a more extensive and specialized knowledge base than less experienced newly graduated physicians and assist them in various aspects of disease management. Some researchers have tested the performance of LLMs using specific clinical questions or guidelines [21,26,28,50], while others have directly inputted clinical case data into the model, allowing it to summarize and provide corresponding diagnostic or treatment decisions autonomously [18,24,45,57,64]. Currently, there is no further research on introducing LLMs into orthopedic operations, likely because of the limited availability of intelligent terminals and digital scenarios that may combine operative procedures with LLMs. Potential docking scenarios for the LLM model could include intelligent surgical applications such as mixed reality operating rooms [89,90] and autonomous laminectomy robots [91,92].

In the context of clinical practice, apart from the fundamental requirement of accurate response, time consumption and work efficiency also serve as crucial reference indicators for evaluating LLMs’ performance. Despite variations in the assessment of model accuracy across the included studies, potentially attributed to differences in research objectives, prompt design, evaluation criteria, and assessment tools, no study has presented evidence indicating that LLMs can independently perform clinical work. Therefore, the current models still require rigorous supervision during their use. An RCT study evaluating the performance of ChatGPT in assisting with orthopedic clinical documentation found that there was no significant efficiency advantage in using ChatGPT: the time taken to complete medical history writing was not superior to voice input, and instances of fabricated content were observed within the ChatGPT-generated medical histories [20].

Although LLMs currently have limitations, they remain valuable tools for orthopedic surgeons in their daily practice. It is important to approach cautiously the responses provided by LLMs and seek additional evidence and explanations from the model used when faced with unclear answers. By incorporating evidence-based medicine tools, we can ultimately achieve superior clinical diagnoses and treatment plans, thereby elevating the quality of care delivered by physicians.

### Application of LLMs in the Field of Research

Research is generally considered a creative endeavor, and introducing LLMs into the field of research may offer more flexibility. Currently, there are limited attempts to use LLMs in orthopedic research. A study found that lowering the reading threshold of professional texts through LLMs can assist in improving the readability of informed consent forms for orthopedic clinical research, but the forms did not meet the recommended sixth-grade reading level set by the American Medical Association [77]. In addition, the literature summarization and generation capabilities of LLMs can contribute to independent or assisted writing of literature reviews in the orthopedic field [79,83]. On the other side of the coin, concerns about integrity arise when scholars find that the model’s output can be deceptively realistic. The abstracts generated by LLMs for studies on *meniscal injuries* and *joint replacement* were indistinguishable from those written by human researchers [78,80]. However, web-based LLMs do not perform well in tasks such as literature review or sample size estimation in sports medicine research [82,84]. Possible reasons may include the potential limitations of LLMs in meeting logical reasoning requirements and the inappropriate use of prompts. For more complex tasks, an optimization approach could involve developing task-specific toolkits based on the fundamental architecture of LLMs. The feasibility of this approach has been validated in interdisciplinary research on the management of back pain [81].

### Application of LLMs in Management

Trained NLP models can convert natural language into structured data and have demonstrated superior performance in tasks involving the current procedural terminology for identifying spinal surgery records [93]. However, ChatGPT, with its larger parameters, performs weaker than NLP models in the task of identifying spinal surgery current procedural terminology codes [85]. One possible reason is that traditional NLP models have been trained on more targeted datasets, whereas researchers cannot fine-tune the backend model of ChatGPT using these data. Despite the current model’s performance limitations hindering its further application in this field, the potential advancements in “fine-tuning” techniques may enable LLMs to assume a more influential role in orthopedic management in the future.

### Current Advantages and Limitations of LLMs in Orthopedic Applications

#### Overview

In contrast to conventional pretrained machine learning models, LLMs exhibit the advantage of versatility by accurately addressing problems across various domains without necessitating additional training on specific samples. In the field of orthopedics, another advantage of LLMs is their user-friendly and convenient nature. Users do not need to go through the long process of waiting and referral from general practitioners to specialists. By simply accessing apps equipped with LLMs, users can inquire about diverse subspecialties in orthopedics at any time and from anywhere, receiving answers promptly at a minimal cost or even free of charge. This service surpasses the capabilities of current health care systems and is unlikely to be replicated in the foreseeable future.

However, as mentioned previously, these advantages are based on unverified answers and unpredictable risks. The answers provided by LLMs for questions related to orthopedics are less robust than those for everyday common knowledge and have significant limitations in terms of accuracy, readability, reliability, and timeliness, as detailed in the following subsections.

#### Accuracy

Almost all studies (66/68, 97%) found errors in LLMs’ responses, with more noticeable inaccuracies in specialized areas such as hip and knee joints and hand surgery [62,67,76]. Some answers even contradicted fundamental orthopedic knowledge [52]. Therefore, some researchers argue that current expectations for guidance provided by AI platforms should be tempered by both physicians and patients [62]. Possible reasons include the limited availability of publicly accessible orthopedic data for training, especially for specialized diseases, as well as privacy concerns that restrict public access to a large amount of data. In the future, besides waiting for more powerful next-generation LLMs, using existing LLMs to learn orthopedic cases and fine-tuning them may be a potential solution to improve accuracy.

#### Readability

Some of the included studies (3/68, 4%) suggest that the content generated by LLMs is not satisfactory in terms of readability for the general population [23,55,58]. The potential reasons for the lack of readability may include not only the limited training data but also the quality of the trained data. By incorporating more popular science content and common clinical responses, it may be possible to address the issue of readability through fine-tuning the model.

#### Reliability

Different ways of asking the same question may yield completely different answers [21]. This instability, particularly in response to specific prompts, not only affects users’ experience and trust but also greatly interferes with researchers’ homogenized evaluations. It is imperative to establish standardized questioning processes and prompt criteria.

#### Timeliness

Training LLMs from scratch is both costly and time consuming, leading to significant retraining expenses. However, unlike everyday common knowledge, orthopedics is constantly evolving with new diagnostic and treatment approaches as well as surgical techniques. Therefore, outdated information becomes an important risk factor leading to inaccurate answers, necessitating caution in this context.

### Methodological Limitations of the Selected Studies

Although there are 47 studies related to clinical issues, only 8 (17%) recruited patients [20,22,24,29,45,48,57,61]. Many of the studies (39/47, 83%) only focus on investigation and evaluation, lacking rigorous methods for clinical research, such as RCTs. Furthermore, there is a lack of research end points directly linked to patient outcomes, such as cure rates or improvements in quality of life, making it difficult to find direct evidence of prognosis. Most of the studies (46/68, 68%) rely on subjective methodologies, such as expert ratings, for model evaluation and lack objective criteria and approaches for assessment, leading to unreliable research results. Furthermore, the absence of standardized questioning paradigms has led to instability in LLM responses, posing challenges for reproducibility and limiting the reliability and clinical significance of the study findings.

### Limitations of This Review

This systematic review has several limitations. First, only English-language articles were included, which may have led to the exclusion of relevant studies published in other languages. Second, due to significant heterogeneity in study designs, model tasks, and evaluation parameters among the included studies, we did not perform a comprehensive synthesis of most of the data, nor did we conduct a meta-analysis. Third, our search was restricted to commonly used medical research databases such as PubMed, Embase, and Cochrane Library, potentially overlooking relevant studies from other sources, including conference papers and gray literature. Finally, given the limited availability of rigorous clinical studies, we included a considerable number of subjective surveys. Although our objective was to provide a broad overview of LLM-related information, this may have introduced bias into the findings. These limitations are expected to be addressed as more standardized, high-quality clinical studies become available in future research.

### Conclusions

Due to the current limitations of LLMs, they cannot replace orthopedic professionals in the short term. However, using LLMs as copilots could be a potential approach to effectively enhance work efficiency at present. In addition, developing task-specific downstream tools based on LLMs is also a potential solution to improve model performance for further use.

## Appendix

Multimedia Appendix 1 PRISMA (Preferred Reporting Items for Systematic Reviews and Meta-Analyses) checklist.

Multimedia Appendix 2 Search strategy.

Multimedia Appendix 3 Data extraction strategy.

Multimedia Appendix 4 Revised Cochrane risk-of-bias tool for randomized trials template for assessment completion.

Multimedia Appendix 5 Quality assessment of studies of large language models based on CONSORT-AI (Consolidated Standards of Reporting Trials–Artificial Intelligence) guidance.

## Abbreviations

AI: artificial intelligence
CONSORT-AI: Consolidated Standards of Reporting Trials–Artificial Intelligence
FKGL: Flesch-Kincaid grade level
GPT: generative pretrained transformer
LLM: large language model
NLP: natural language processing
OITE: Orthopaedic Surgery In-Training Examination
PGY: postgraduate year
PLM: pretrained language model
PRISMA: Preferred Reporting Items for Systematic Reviews and Meta-Analyses
RCT: randomized controlled trial

## Data Availability

All data generated or analyzed during this study are included in this published paper and its supplementary information files.
