# Supplementary material for: Examining the Role of Large Language Models in Orthopedics: Systematic Review
Source: J Med Internet Res. 2024 Nov 15;26:e59607. doi: 10.2196/59607 (PMC11607553; doi:10.2196/59607)
Supplement: Multimedia Appendix 2 [file jmir_v26i1e59607_app2.docx]

**Appendix 2** Search strategy

**Pubmed**

("large language model" OR "large language models" OR "LLM" OR "LLMs" OR "generative artificial intelligence" OR "Generative AI" OR "ChatGPT" OR "Generative Pre-Trained Transformer") AND ((((((((((((((((((((Orthopaedics) OR (bone)) OR (musculoskeletal)) OR (injury)) OR (wound)) OR (trauma)) OR (articular)) OR (joint)) OR (sports medicine)) OR (hand surgery)) OR (spine)) OR (spinal)) OR (cervical vertebrae)) OR (thoracic vertebrae)) OR (lumbar vertebrae)) OR (sacrum)) OR (coccyx)) OR (spinal canal)) OR (vertebral body)) OR (intervertebral disc))

**Embase**

#1

('large language model' OR 'llm' OR 'generative artificial intelligence' OR 'generative ai' OR 'chatgpt' OR 'generative pre-trained transformer')

#2

('orthopaedics' OR 'bone' OR 'musculoskeletal' OR 'injury' OR 'wound' OR 'trauma' OR 'articular' OR 'joint' OR 'sports medicine' OR 'hand surgery' OR 'spine' OR 'spinal' OR 'cervical vertebrae' OR 'thoracic vertebrae' OR 'lumbar vertebrae' OR 'sacrum' OR 'coccyx' OR 'spinal canal' OR 'vertebral body' OR 'intervertebral disc')

#3

#1 AND #2

**Cochrane**

#1 (orthopaedics):ti,ab,kw

#2 (Articular):ti,ab,kw

#3 (joint):ti,ab,kw

#4 (bone):ti,ab,kw

#5 (musculoskeletal):ti,ab,kw

#6 (injury):ti,ab,kw

#7 (wound):ti,ab,kw

#8 (trauma):ti,ab,kw

#9 (sports medicine):ti,ab,kw

#10 (hand surgery):ti,ab,kw

#11 (spine):ti,ab,kw

#12 (spinal):ti,ab,kw

#13 (Cervical Vertebrae):ti,ab,kw

#14 (Thoracic Vertebrae):ti,ab,kw

#15 (Lumbar Vertebrae):ti,ab,kw

#16 (Sacrum):ti,ab,kw

#17 (Coccyx):ti,ab,kw

#18 (Spinal Canal):ti,ab,kw

#19 (Vertebral Body):ti,ab,kw

#20 (Intervertebral Disc):ti,ab,kw

#21 #1 OR #2 OR #3 OR #4 OR #5 OR #6 OR #7 OR #8 OR #9 OR #10 OR #11 OR #12 OR #13 OR #14 OR #15 OR #16 OR #17 OR #18 OR #19 OR #20

#22 (large language model):ti,ab,kw

#23 (LLM):ti,ab,kw

#24 (generative artificial intelligence):ti,ab,kw

#25 (Generative AI):ti,ab,kw

#26 (ChatGPT):ti,ab,kw

#27 (Generative Pre-Trained Transformer):ti,ab,kw

#28 #22 OR #23 OR #24 OR #25 OR #26 OR #27

#29 #21 AND #28 in Trials
