# Supplementary material for: Examining the Role of Large Language Models in Orthopedics: Systematic Review
Source: J Med Internet Res. 2024 Nov 15;26:e59607. doi: 10.2196/59607 (PMC11607553; doi:10.2196/59607)
Supplement: Multimedia Appendix 3 [file jmir_v26i1e59607_app3.docx]

**Appendix 3.** Data extraction strategy

**General** **characteristics**

- Title
- Author, year
- Study design,
- Tasks
- LLM tools

**LLM for clinical practice**

- Main evaluation metrics for model performance and their values
- Number of enrolled participants
- Subjective or objective evaluation of model performance

**LLM for orthopaedic education**

- Source of the test questions
- Number of questions
- Scores/accuracy

**LLM for scientific research and management**

- Model input
- Key findings
