# Supplementary material for: Examining the Role of Large Language Models in Orthopedics: Systematic Review
Source: J Med Internet Res. 2024 Nov 15;26:e59607. doi: 10.2196/59607 (PMC11607553; doi:10.2196/59607)
Supplement: Multimedia Appendix 5 [file jmir_v26i1e59607_app5.docx]

**Appendix 5.** Quality assessment on LLMs studies based on CONSORT guidance - AI extension

|  | Study | Statement of AI algorithm | Use of AI intervention in context of the clinical pathway | Inclusion and exclusion criteria at the level of the input data | Description of the approaches to handle unavailable input data | Describe input data acquisition process for AI intervention | Specifications of human-AI interaction in the collection of input data | Output of the AI algorithm | Explanations of how AI intervention’s outputs contribute to health behavior changes |  |
| --- | --- | --- | --- | --- | --- | --- | --- | --- | --- | --- |
| 1 | Coraci et al [1], 2023 | + | + | + | NR | + | NR | + | + |  |
| 2 | Daher et al [2], 2023 | + | + | + | NR | + | NR | + | + |  |
| 3 | Fabijan et al [3], 2023 | + | + | + | NR | + | + | + | + |  |
| 4 | Pagano et al [4], 2023 | + | + | + | NR | + | + | + | + |  |
| 5 | Schonfeld et al [5], 2024 | + | + | + | NR | + | + | + | + |  |
| 6 | Truhn et al [6], 2023 | + | + | + | NR | + | + | + | + |  |
| 7 | Yang et al [7], 2024 | + | + | + | NR | + | NR | + | + |  |
| +  NR | Described  Not reported | | | | | | | | | |

**References**

1. Coraci D, Maccarone MC, Regazzo G, Accordi G, Papathanasiou JV, Masiero S. ChatGPT in the development of medical questionnaires. The example of the low back pain. European Journal of Translational Myology. 2023;33(4). doi: 10.4081/ejtm.2023.12114.

2. Daher M, Koa J, Boufadel P, Singh J, Fares MY, Abboud JA. Breaking barriers: can ChatGPT compete with a shoulder and elbow specialist in diagnosis and management? JSES International. 2023;7(6):2534-41. doi: 10.1016/j.jseint.2023.07.018.

3. Fabijan A, Polis B, Fabijan R, Zakrzewski K, Nowosławska E, Zawadzka-Fabijan A. Artificial Intelligence in Scoliosis Classification: An Investigation of Language-Based Models. Journal of Personalized Medicine. 2023;13(12). doi: 10.3390/jpm13121695.

4. Pagano S, Holzapfel S, Kappenschneider T, Meyer M, Maderbacher G, Grifka J, Holzapfel DE. Arthrosis diagnosis and treatment recommendations in clinical practice: an exploratory investigation with the generative AI model GPT-4. Journal of Orthopaedics and Traumatology. 2023;24(1). doi: 10.1186/s10195-023-00740-4.

5. Schonfeld E, Pant A, Shah A, Sadeghzadeh S, Pangal D, Rodrigues A, et al. Evaluating Computer Vision, Large Language, and Genome-Wide Association Models in a Limited Sized Patient Cohort for Pre-Operative Risk Stratification in Adult Spinal Deformity Surgery. J Clin Med. 2024 Jan 23;13(3). PMID: 38337352. doi: 10.3390/jcm13030656.

6. Truhn D, Weber CD, Braun BJ, Bressem K, Kather JN, Kuhl C, Nebelung S. A pilot study on the efficacy of GPT-4 in providing orthopedic treatment recommendations from MRI reports. Sci Rep. 2023 Nov 17;13(1):20159. PMID: 37978240. doi: 10.1038/s41598-023-47500-2.

7. Yang F, Yan D, Wang Z. Large-Scale assessment of ChatGPT's performance in benign and malignant bone tumors imaging report diagnosis and its potential for clinical applications. J Bone Oncol. 2024 Feb;44:100525. PMID: 38314324. doi: 10.1016/j.jbo.2024.100525.
